# Antioxidative and Cardioprotective Effects of *Schisandra chinensis* Bee Pollen Extract on Isoprenaline-Induced Myocardial Infarction in Rats

**DOI:** 10.3390/molecules24061090

**Published:** 2019-03-20

**Authors:** Zhenhuang Shen, Qianqian Geng, Haibo Huang, Hong Yao, Tianyu Du, Lifu Chen, Zhenhong Wu, Xiaoqing Miao, Peiying Shi

**Affiliations:** 1 Bee Science College, Fujian Agriculture and Forestry University, Fuzhou 350002, China; 18065021034@163.com (Z.S.); qqgengfafu@163.com (Q.G.); haibohuang329@163.com (H.H.); tianyu1988339@sina.com (T.D.); clfbee@126.com (L.C.); wzh516@126.com (Z.W.); 2 College of Food Science, Fujian Agriculture and Forestry University, Fuzhou 350002, China; 3 State and Local Joint Engineering Laboratory of Natural Biotoxins, Fujian Agriculture and Forestry University, Fuzhou 350002, China; 4 Department of Pharmaceutical Analysis, School of Pharmacy, Fujian Medical University, Fuzhou 350122, China; yauhung@126.com

**Keywords:** *Schisandra chinensis* bee pollen extract, myocardial infarction, cardioprotective effect, antioxidative effect, anti-apoptotic effect

## Abstract

Oxidative stress plays an important role in the pathogenesis of myocardial infarction (MI). *Schisandra chinensis* bee pollen extract (SCBPE) possesses powerful antioxidant capacity. This study aimed to further explore the antioxidative and cardioprotective effects of SCBPE on acute MI induced by isoprenaline (ISO) in rats. The rats were intragastrically administrated with SCBPE (600, 1200, or 1800 mg/kg/day) and Compound Danshen dropping pills (270 mg/kg/day) for 30 days, then subcutaneously injected with ISO (65 mg/kg/day) on the 29th and 30th day. Compared with the model group, pretreatment with middle and high doses of SCBPE significantly reduced serum aspartate transaminase, lactate dehydrogenase, and creatine kinase activities and increased myocardial superoxide dismutase, glutathione peroxidase, and catalase activities. The histopathologic aspects showed that pathological heart change was found in the model group and reduced to varying degrees in the SCBPE groups. Moreover, the protein expression of nuclear factor-erythroid 2-related factor 2 (Nrf-2), heme oxygenase-1 (HO-1), and Bcl2 in the heart increased in the SCBPE groups, while that of Bax decreased compared to the model group. Besides this, uridine was isolated from *S. chinensis* bee pollen for the first time. This study could provide a scientific basis for using *Schisandra chinensis* bee pollen as a functional food for the prevention of MI.

## 1. Introduction

Myocardial infarction (MI) is a major cause of death and disability worldwide; it may be the first manifestation of coronary artery disease or it may occur repeatedly in patients with established disease [1]. It is generally acknowledged that oxidative stress plays an important role in the pathogenesis of MI. Overproduction of reactive oxygen species (ROS) may affect cell membrane properties and cause oxidative damage to nucleic acids, lipids, and proteins that may make them nonfunctional. [2]. Thus, therapeutic treatment with antioxidants may be a potent strategy to prevent cardiac damage and myocardial dysfunction in patients who suffer from acute MI [3].

Isoprenaline (ISO), a synthetic catecholamine and β-adrenergic receptor agonist, has been reported to induce infarct-like myocardial lesions under subcutaneous administration [4]. The cardiotoxicity induced by high levels of ISO is mediated by vasospasticity, combined with increased oxygen demand due to its positive inotropic effect, and is also related to the oxidative stress [5,6,7]. Accordingly, ISO-induced MI is now commonly used as a well-standardized nonsurgical model to evaluate the protective effects of potential medicines [8,9,10].

Increasing attentions have been focused on the cardioprotective effect of natural products from medicinal and edible plants, such as total flavonoids from *Clinopodium chinense* (Benth.) O. Ktze [11], *Astragalus* polysaccharide [12], seabuckthorn pulp oil [13], and linseed oil [14], etc. In addition, several herbal medicines, such as *Nepeta deflersiana*, have demonstrated antioxidant, anti-inflammatory, and anti-apoptotic capacities against MI induced by ISO [15].

Bee pollen, which is the material that adheres to honeybees during collection, is obtained from collected flower pollen and nectar, agglutinated into balls with bee saliva substances [16]. Due to its numerous species of nutritional and bioactive compounds, bee pollen has been reported to possess many pharmacological properties, including antioxidant [17], antimicrobial [17], anti-inflammatory [18], hepatoprotective [19], and anticarcinogenic effects [20], and so on. Besides this, bee pollen also has cardiovascular protective effects. For example, enzymatic hydrolysates from honeybee-collected pollen of *Cistus ladaniferus* showed high angiotensin I-converting enzyme inhibitory activities in the body system, suggesting potential antihypertensive activities [21].

*Schisandra chinensis* bee pollen has been widely accepted as a functional food in China for several decades. Studies have confirmed its powerful antioxidant capacity among 14 species of monofloral bee pollen from China [22]. Besides this, *S. chinensis* bee pollen has also been reported to possess the strongest total antioxidant capacity among ten kinds of bee pollens based on Trolox equivalent antioxidant capacity, reducing power, and DPPH• radical scavenging activity [23]. Owing to its favorable antioxidative properties, SCBPE is also reasonably deduced to possess a protective effect against MI. In addition, the active components in SCBPE are little known. All these suggest that further investigation is required on the protective effect and substance constituents of SCBPE for the sake of the development and usage of SCBPE in the field of health care.

In this study, first, the components of SCBPE were analyzed by HPLC and the antioxidant activity in vitro was further analyzed. Then, the cardioprotective effects of SCBPE on acute MI induced by ISO in rats were validated and investigated by evaluating serum cardiac enzyme levels, myocardial antioxidant enzyme activities, and histopathological changes, combined with the protein expression of nuclear factor-erythroid 2-related factor 2 (Nrf-2), heme oxygenase-1 (HO-1), Bcl-2-associated x (Bax), and Bcl-2 in heart tissues using Western blotting.

## 2. Results

### 2.1. HPLC Analysis and Antioxidant Activity Analysis In Vitro

**Figure 1:**
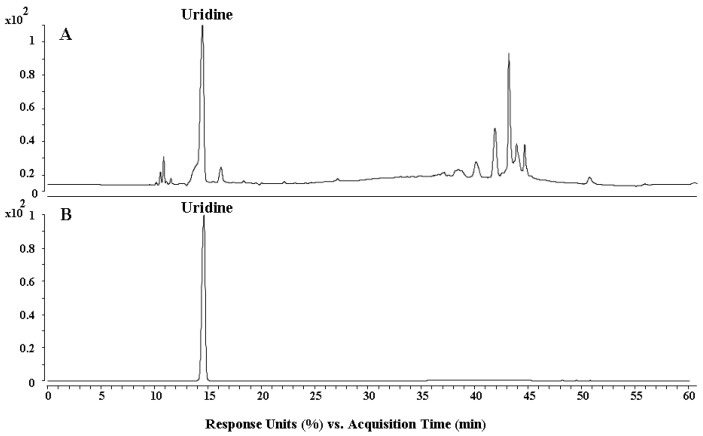
HPLC chromatograms of *Schisandra chinensis* bee pollen extract (SCBPE) (**A**) and a uridine standard (**B**).

The components of SCBPE were analyzed by HPLC. As shown in Figure 1, one major compound was identified as uridine by comparison to its standard reference, appearing with double peaks at 10.99 and 11.71 min, which was isolated from *S. chinensis* bee pollen for the first time.

In addition, the antioxidant activity in vitro was also analyzed. The capacities of 2,2′-azinobis-(3-ethylbenzthiazoline-6-sulphonate) (ABTS^+•^) scavenging activity and ferric-reducing antioxidant power (FRAP) were 0.37 ± 0.01 and 0.33 ± 0.01 mM TE·g^−1^, respectively. From the results, it can be inferred that SCBPE possesses ABTS^+•^ radical scavenging activity and ferric-reducing antioxidant activity in vitro.

### 2.2. Effects of SCBPE on Serum Cardiac Enzymes in MI Rats Induced by ISO

The serum cardiac enzymes among the experimental groups are all summarized in Table 1. The levels of aspartate transaminase (AST), lactate dehydrogenase (LDH), and creatine kinase (CK) in the model group were all dramatically increased compared with those in the control group (*p* < 0.01), evidently indicating that administration of ISO caused MI in rats. Compared to the model group, pretreatment with Compound Danshen dropping pills (CDDP) and a middle dose of SCBPE significantly reduced the levels of AST, LDH, and CK (*p* < 0.05) in serum, and administration of a high dose of SCBPE significantly reduced the levels of the four serum cardiac enzymes (*p* < 0.01). In addition, pretreatment with SCBPE significantly reduced the elevated serum cardiac enzymes in MI rats induced by ISO in a dose-dependent manner.

### 2.3. Effects of SCBPE on Myocardial Antioxidant Enzyme Activities in MI Rats Induced by ISO

As depicted in Table 2, compared with in the control group, the activities of myocardial glutathione peroxidase (GSH-Px), superoxide dismutase (SOD), and catalase (CAT) in the model group declined significantly (*p* < 0.01). Compared to in the model group, the levels of myocardial SOD, GSH-Px (*p* < 0.01), and CAT (*p* < 0.05) were significantly increased in the CDDP and middle-dose SCBPE groups, and the activities of the three myocardial antioxidant enzymes (*p* < 0.01) were significantly increased in the high-dose SCBPE group. In addition, with the increase of the pretreatment SCBPE dose, the activities of the myocardial antioxidant enzymes in MI rats were increased in a dose-dependent manner.

### 2.4. Effects of SCBPE on the Pathological Changes of Rat Hearts

**Figure 2:**
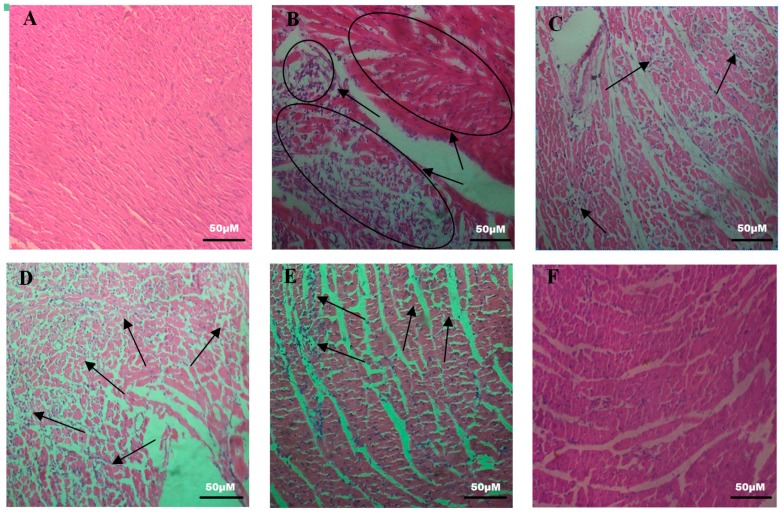
Effects of SCBPE on pathological changes in the heart in rats (200×, HE). (**A**) control group; (**B**) model group; (**C**) CDDP group; (**D**) 600 mg/kg of SCBPE group; (**E**) 1200 mg/kg of SCBPE group; (**F**) 1800 mg/kg of SCBPE group.

According to the hematoxylin-eosin (HE) staining of the heart tissue (Figure 2), normal histology of the heart tissue with striations, a branched appearance, and continuity with adjacent myofibrils was observed in the control group. In the model group, cardiac muscle fiber was disorganized with edema, inflammatory infiltration, and inferior continuity with adjacent myofibrils. The damage to the SCBPE administration groups was lower than that to the model group, and the high-dose SCBPE group showed the lowest inflammatory infiltration among all the SCBPE groups and almost normal cardiac fibers.

### 2.5. Effects of SCBPE on the Protein Expression of Nrf-2 and HO-1 in Heart Tissues of MI Rats Induced by ISO

**Figure 3:**
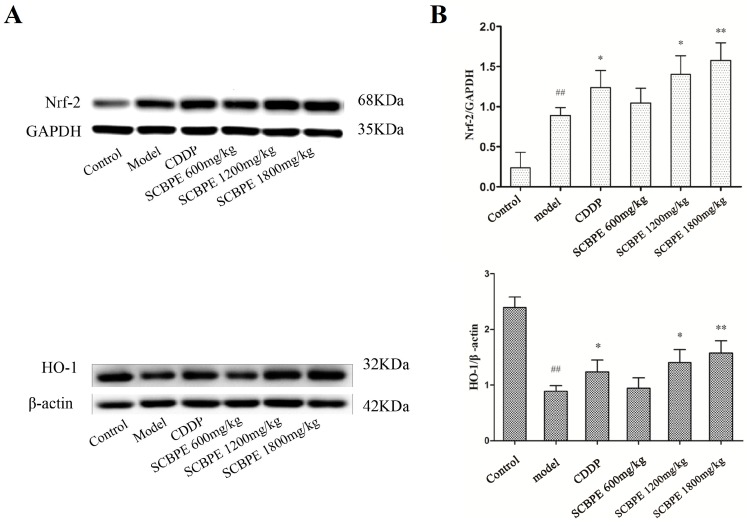
The effects of SCBPE on the protein expression of Nrf-2 and HO-1 in heart tissues (*n* = 3). (**A**) The protein expression of Nrf-2 and HO-1 in heart tissues by Western blot; GAPDH and β-actin were used as internal control. (**B**) The respective ratios of the protein expression levels of Nrf-2 and HO-1 to the internal control. ^##^ Model group vs. control group, *p* < 0.01; * CDDP and SCBPE groups vs. model group, *p* < 0.05; ** CDDP and SCBPE groups vs. model group, *p* < 0.01.

The effects of SCBPE on the protein expression of Nrf-2 and HO-1 in heart tissues are shown in Figure 3. Compared with the control group, the protein expression of Nrf-2 in heart tissues in the model group was significantly elevated (*p* < 0.01), while the protein expression of HO-1 was significantly decreased (*p* < 0.01). Compared to the model group, the protein expression of Nrf-2 and HO-1 in heart tissues was significantly increased in the CDDP and middle-dose SCBPE groups (*p* < 0.05), and it was significantly increased in the high-dose SCBPE group (*p* < 0.01). Moreover, with the increase of the pretreatment SCBPE dose, the protein expression of Nrf-2 and HO-1 in MI rats was enhanced in a dose-dependent manner.

### 2.6. Effects of SCBPE on the Protein Expression of Bax and Bcl-2 in Heart Tissues of MI Rats Induced by ISO

**Figure 4:**
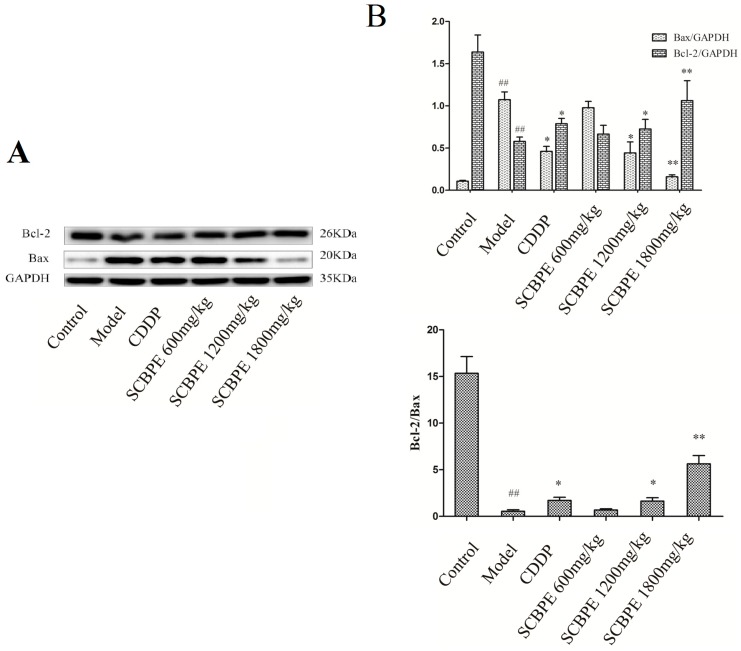
The effects of SCBPE on the protein expression of Bax and Bcl-2 in heart tissues (*n* = 3). (**A**) The protein expression of Bax and Bcl-2 in heart tissues by Western blot; GAPDH was used as internal control. (**B**) The respective ratios of the protein expression of Bax and Bcl-2 to the internal control and the ratio of the protein expression of Bcl-2 to that of Bax. ^##^ Model group vs. control group, *p* < 0.01; * CDDP and SCBPE groups vs. model group, *p* < 0.05; ** CDDP and SCBPE groups vs. model group, *p* < 0.01.

The effects of SCBPE on the protein expression of Bax and Bcl-2 in heart tissues are shown in Figure 4. Compared with the control group, the protein expression of Bax in heart tissues in the model group was significantly elevated (*p* < 0.01), while the protein expression of Bcl-2 and the ratio of Bcl-2 to Bax were significantly decreased (*p* < 0.01), suggesting that large-scale apoptosis occurred in rat cardiac myocytes. Compared to the model group, the protein expression of Bax in heart tissues was significantly reduced in the CDDP and middle-dose SCBPE groups (*p* < 0.05), and it was significantly decreased in the high-dose SCBPE group (*p* < 0.01). On the contrary, pretreatment with CDDP and a middle dose of SCBPE significantly increased the protein expression of Bcl-2 and the ratio of Bcl-2 to Bax in heart tissues; further, the administration of a high dose of SCBPE significantly increased the protein expression of Bcl-2 and the ratio of Bcl-2 to Bax (*p* < 0.01).

## 3. Discussion

Injected ISO could cause destruction and damage to the myocardial cell membrane [4]. Thereafter, the cardiac enzymes leak out from damaged tissues to the blood stream, and the levels of cardiac enzymes in blood abnormally increase; this, in turn, represents the plasma membrane integrity and/or permeability and serves as a diagnostic marker to evaluate the severity of MI [24,25]. In the present study, the levels of AST, LDH, and CK in serum were dramatically elevated in rats treated with ISO compared with the control group, indicating that administration of ISO caused MI in rats. Pretreatment with SCBPE significantly decreased the levels of the four cardiac enzymes in serum, suggesting that SCBPE could reduce the injury caused by ISO to myocardial cells and protect the integrity and permeability of the cell membrane. In addition, the pathological changes of hearts in rats also suggested that the administration of SCBPE could effectively reduce the cardiotoxicity caused by ISO. However, the limitation of our study is that cardiac troponin I and T were not detected; these are components of the contractile apparatus of myocardial cells, are expressed almost exclusively in the heart, and serve as the preferred biomarkers for myocardial necrosis [1].

Furthermore, the components of SCBPE were analyzed by HPLC, and uridine was isolated from *S. chinensis* bee pollen for the first time. Besides this, the antioxidant activity analysis in vitro showed that SCBPE possessed strong antioxidant capacity, which could have a great contribution to the observed anti-MI effect.

ISO-induced MI is generally attributed to the formation of the highly reactive hydroxyl radical (OH·), a stimulator of lipid peroxidation and source of destruction and damage to cell membranes [26]. Antioxidant enzymes such as SOD, CAT, and GSH-Px serve as the first line of the cellular defense system in preventing oxidative stress by stimuli [27]. In our results, the activities of myocardial SOD, CAT, and GSH-Px were decreased significantly in the model group compared to in the control group. The reduction in the activities of SOD and CAT in ISO-induced MI may be due to the increased generation of reactive oxygen radicals, such as superoxide and hydrogen peroxide, which in turn leads to the inhibition of activities of these enzymes [28]. Suppression of GSH-Px leads to the accumulation of ROS and makes the cardiomyocytes more susceptible to oxidative injury [29]. On the contrary, pretreatment with middle and high doses of SCBPE significantly enhanced the activities of those enzymes, displaying antioxidant system enhancement by SCBPE. These data confirmed that the cardioprotective effects of SCBPE are greatly related to its antioxidative activities.

Nrf-2 could regulate the expression of a battery of antioxidant enzymes and phase 2 detoxifying enzymes such as SOD, GSH-Px, CAT, and quinone oxidoreductase-1 in cardiac cells in order to prevent injury caused by free radicals [30]. Thus, Nrf-2 is recognized as a critical component involved in the induction of antioxidative genes which could provide protection against oxidative-stress-induced damage in cardiovascular diseases [31]. In our results, with pretreatment with SCBPE, the up-regulation of SOD, GSH-Px, and CAT in rat heart under the control of Nrf2 might be an adaptive mechanism to overcome the ISO-induced oxidative stress and MI in rats. In addition, Nrf-2 translocation has been further demonstrated to regulate antioxidant response by playing an essential role in the induction of HO-1 [32]. HO-1 could catalyze the degradation of heme to yield biliverdin, carbon monoxide, and iron, the induction of which could be important for the survival and ultimate healing of cardiac structures after experimental myocardial infarction [33], and HO-1 is associated with a lower severity of coronary artery disease [34]. In the present study, we observed a significant increase in myocardial HO-1 expression in SCBPE-pretreated rats compared with in the model group. In addition, the multipathway capability of SCBPE to reduce the burden of lipid peroxidation and tissue damage is consistent with recent findings by Locatelli et al., which revealed the protective effects of Graminex pollen on rat prostate challenged with LPS through its down-regulating effects on the pro-inflammatory NFκB pathway [35].

It is well acknowledged that necrosis and apoptosis are both involved in cardiocyte death in acute MI [36]. Anti-apoptosis and pro-apoptosis protein members of the Bcl-2 family act as regulators of cellular fate by integrating different death or survival signal pathways [37]. As members of the Bcl-2 family, Bax promotes apoptosis and Bcl-2 blocks apoptosis. The ratio of Bcl-2 to Bax could determine survival or death following an apoptotic stimulus [38]. ROS can trigger myocyte apoptosis by up-regulating proapoptotic proteins such as Bax, and these apoptotic proteins are inhibited by various antioxidants as well as by overexpression of the antiapoptotic protein Bcl-2 [39]. In our study, the protein expression of Bcl-2 and the ratio of Bcl-2 to Bax in heart tissues were increased, and the protein expression of Bax was reduced with pretreatment with middle and high doses of SCBPE compared with the model group. These data indicated that pretreatment with SCBPE could inhibit cardiocyte apoptosis through regulating Bax and Bcl-2, thus providing an anti-apoptosis strategy against MI damage in rats.

## 4. Material and Methods

### 4.1. Chemicals and Reagents

*S. chinensis* bee pollen was purchased from Xuchang (*Lon*, *Lat*: 113.835312,34.02674), Henan, China in October, 2015, and identified according to China national standard GB/T 30359-2013. Isoprenaline hydrochloride, ABTS, 2,4,6-tris(2′-pyridyl)-*s*-triazine (TPTZ), and 6-hydroxy-2,5,7,8-tetramethylchroman-2-carboxylic acid (Trolox) were obtained from Aladdin Reagent Co., Ltd. (Shanghai, China). Pentobarbital sodium was purchased from Sigma-Aldrich Co, St Louis, MO, USA. CDDP were purchased from local drugstores.

### 4.2. Preparation of SCBPE

*S. chinensis* bee pollen samples were dried at 37 °C and pulverized into powder, then reflux-extracted twice with 70% ethanol at a ratio of 1:15 (*w*/*v*) for 2 h. After filtration, the extract was centrifuged at 3300× *g* for 15 min. Then, the supernatant was collected, concentrated at 45 °C and 0.05 MPa, and freeze-dried for 36 h to obtain the SCBPE.

### 4.3. HPLC Analysis

For clarifying the main ingredients, 500 milligrams of SCBPE were dissolved in 10 mL of 70% methanol. The sample was then centrifuged at 13000 rpm for 15 min and the supernatant was filtered through a 0.45 μm filter. The separation of the components was carried out using an Agilent 1290 Infinity LC instrument (Agilent, Waldbronn, Germany) consisting of a binary pump, a diode-array detector, an auto-sampler, and a column compartment. The samples were separated on a YMC-pack ODS-A column (10 × 250 mm, 5 μm), and on-line UV spectra were recorded at the wavelength of 254 nm. The separation procedures were set as water–methanol with gradient elution for 30 min. The flow rate was 1 mL/min, and the injection volume was 18 μL. As a result, one main component was isolated by HPLC and identified as uridine according to the UV, MS, ^1^H NMR, and ^13^C NMR data (see Supplementary Materials).

Ultimately, the main components contained in SCBPE were analyzed using an Ultimate XB-C_18_ column, 5 μm, 250 mm × 4.6 mm i.d. (Welch Materials, Inc., Ellicott, MD, USA), and the mobile phase was a stepwise gradient of water (containing 0.1% formic acid, *v*/*v*) and methanol (0 min, 80:20; 30 min, 35:65; 31–50 min, 20:80; 50–60 min, 10:90). The flow rate was 0.25 mL/min, and the injection volume was 5 μL.

### 4.4. Analysis of Antioxidant Capacity

#### 4.4.1. ABTS Radical Cation Scavenging Activity

The ABTS radical cation scavenging activity of the extract was analyzed by a colorimetric method described by Re et al. with some modifications [40]. A volume of 176 μL of 140 mM potassium persulfate solution was reacted with 10 mL of 0.7 mM ABTS solution at room temperature in the dark for 13–14 h to prepare the ABTS radical cation (ABTS^+•^) solution. Then, the ABTS^+•^ solution was diluted with ethanol until the absorbance was 0.700 ± 0.02 at 734 nm before use. Afterwards, 0.2 mL of the sample was mixed with 3.8 mL of diluted ABTS^+•^ solution and allowed to stand at room temperature in the dark for 10 min. The absorbance was measured at 734 nm. Trolox was used as a standard to construct the calibration curve, and the ABTS radical cation scavenging activity was expressed as millimole Trolox equivalents per gram of extract (mmol TE·g^−1^).

#### 4.4.2. FRAP Assay

FRAP assay was performed according to the instructions of Benzie and Strain with some modifications [41]. FRAP working solution was prepared by mixing 300 mM acetate buffer (pH = 3.6) with 10 mM TPTZ and 20 mM ferric chloride (FeCl_3_) in the ratio of 10:1:1. A volume of 0.2 mL of sample was mixed with 3.8 mL of FRAP working solution under a water bath at 37 °C in the dark for 10 min. The absorbance was determined at 593 nm against the blank. Similar to the ABTS^+•^ scavenging assay, Trolox was used as a standard, and the antioxidant capacity of the FRAP assay was expressed as millimole Trolox equivalents per gram of extract (mmol TE·g^−1^).

### 4.5. Animal Experiments

#### 4.5.1. Experimental Animals

Animal experiments were approved by the Animal Care and Use Committee of the College of Animal Science, Fujian Agriculture and Forestry University (Certification Number: CNFJAC0027), and the experiment was performed according to the regulations and guidelines established by this committee. Thirty-six healthy male Sprague Dawley rats weighing from 200 to 230 g were obtained from the Wushi Laboratory Animal Center, Fuzhou. Rats were housed at controlled temperature (25 ± 2 °C) and humidity (55 ± 10%) with a 12 h light and dark cycle, and they had free access to commercial rat food and filtered tap water.

#### 4.5.2. Experimental Design

After feeding adaptively for 7 days, the rats were randomly allocated into six groups of six rats as follows:

Group 1 (*n* = 6) served as the control group, and these rats were intragastrically administrated with normal saline daily for 30 days;

Group 2 (*n* = 6) served as the model group, and these rats were intragastrically administrated with normal saline daily for 30 days and subcutaneously injected with ISO at a dose of 65 mg/kg/day on the 29th and 30th days;

Group 3 (*n* = 6) was intragastrically administrated with CDDP suspension prepared with normal saline at a dosage of 270 mg/kg/day for 30 days and subcutaneously injected with ISO at a dose of 65 mg/kg/day on the 29th and 30th days;

Groups 4, 5, and 6 (*n* = 6 in each) were intragastrically administrated with SCBPE suspensions prepared with normal saline (600, 1200, and 1800 mg/kg/day, respectively) for 30 days and subcutaneously injected with ISO (65 mg/kg/day) on the 29th and 30th days.

Then, after an overnight fast and free access to water, rats were all anaesthetized with an intraperitoneal injection of pentobarbital sodium (50 mg/kg) and sacrificed. Blood samples were harvested from the abdominal aorta using blood collection tubes. Heart tissues were removed, washed in ice-cold isotonic saline, dried with filter paper, collected in containers, and kept in liquid nitrogen for further study.

#### 4.5.3. Biochemical Assay in the Serum

Serum samples were separated by centrifugation at 4 °C at the speed of 3000 rpm for 10 min. The myocardial enzyme levels of AST, LDH, and CK in serum were determined using an AU2700 biochemical analyzer (Olympus, Japan) in Air Force Fuzhou Hospital.

#### 4.5.4. Determination of Antioxidant Parameters in Myocardial Tissues

Prior to determinations, heart tissue samples were thawed at 4 °C. Homogenates (10%) were prepared using phosphate buffer solution (pH = 7.4) and centrifuged at 3500× *g* for 15 min at 4 °C. The resulting supernatant was used for the determination of SOD, GSH-Px, CAT, and protein concentrations using commercial diagnostic kits (Nanjing Jiancheng Bioengineering Institute, Nanjing, China). The activities of SOD, GSH-Px, and CAT were normalized with the protein concentration according to the manufacturer’s protocol.

#### 4.5.5. Histopathological Examinations

The heart tissues were rapidly dissected out and fixed in 10% neutral formalin solution for 24 h and embedded in paraffin. For histological examinations, sections of 5 μm in thickness were cut and stained with haematoxylin and eosin (H & E). Observation of structural abnormality was performed under optical microscopy (Chongqing Optec Instrument, model: B203LED) at 200× magnification equipped with an Optec DV500 digital camera.

#### 4.5.6. Western Blotting Analyses

The thawed samples were homogenized in total protein extraction buffer with added proteinase inhibitor. After determination of the protein concentration, denatured protein was subjected to 10% SDS-PAGE and transferred to polyvinylidene fluoride membranes (Millipore Corporation, Billerica, MA, USA). After blocking, the membranes were probed with rabbit polyclonal antibodies anti-Nrf2 (Santa Cruz Biotechnology, Santa Cruz, CA, USA), anti-HO-1 and anti-Bcl-2 (Abcam, UK), anti-Bax (CST, Danvers, MA, USA), anti-GAPDH (Proteintech Group, Inc, Wuhan, China), and anti-β-actin (Guge, Wuhan, China) at 4 °C overnight, followed by horseradish-peroxidase-conjugated secondary anti-rabbit antibodies (Proteintech Group, Inc, China) at room temperature for 1 h. The specific bands were visualized with electrochemiluminescence (ECL) reagent and captured by G:BOX Chemi XT4 (Syngene, Frederick, MD, USA). For quantification, the integral optical density (IOD) values of Nrf2, HO-1, Bcl-2, Bax, GAPDH, and β-actin were analyzed using Image-Pro Plus 6.0 software (Media Cybernetics Inc, Silver Spring, MD, USA).

### 4.6. Statistical Analysis

All values are expressed as mean ± SD and were analyzed by one-way analysis of variance (ANOVA) followed by Least Significant Difference (LSD) among groups using Statistical Product and Service Solutions (SPSS) 17.0 software package for Windows (IBM, USA). Values of *p* less than 0.05 were considered to indicate statistically significant differences between groups.

## 5. Conclusions

This study revealed that SCBPE could exert antioxidative and cardioprotective effects on ISO-induced MI in rats. Besides this, uridine was isolated from *S. chinensis* bee pollen for the first time. Collectively, the positive effect of SCBPE confirmed in this study could provide a scientific basis for using *Schisandra chinensis* bee pollen as a functional food for the prevention of MI.

## Figures and Tables

**Table 1:** Effects of SCBPE on serum cardiac marker enzymes in myocardial infarction (MI) rats induced by isoprenaline (ISO) (*n* = 6).

Group	AST (U/L) ^a^	LDH (U/L) ^a^	CK (U/L) ^a^
Control group	87.00 ± 8.10	423.33 ± 129.04	382.83 ± 121.11
Model group	284.30 ± 79.28 ^##^	1468.40 ± 416.06 ^##^	954.20 ± 353.05 ^##^
CDDP group	145.50 ± 60.26 *	856.50 ± 282.77 *	580.67 ± 218.88 *
SCBPE (600 mg/kg) group	254.80 ± 96.90	919.60 ± 314.29	757.20 ± 279.17
SCBPE (1200 mg/kg) group	163.80 ± 73.09 *	771.33 ± 166.13 *	604.17 ± 191.42 *
SCBPE (1800 mg/kg) group	146.3 ± 63.13 **	560.00 ± 187.45 **	409.00 ± 128.36 **

^a^ Values represent mean ± SD of six animals; ^##^ Model group vs. control group, *p* < 0.01; * CDDP and SCBPE groups vs. model group, *p* < 0.05; ** CDDP and SCBPE groups vs. model group, *p* < 0.01.

**Table 2:** Effects of SCBPE on myocardial antioxidant enzyme activities in MI rats induced by ISO (*n* = 6).

Group	SOD (U/mg prot) ^a^	GSH-Px (U/mg prot) ^a^	CAT (U/mg prot) ^a^
Control group	263.92 ± 15.69	123.72 ± 13.02	13.77 ± 1.04
Model group	155.53 ± 17.71 ^##^	78.74 ± 8.78 ^##^	7.87 ± 1.59 ^##^
CDDP group	203.39 ± 19.48 **	100.62 ± 4.95 **	10.62 ± 1.34 *
SCBPE (600 mg/kg) group	175.25 ± 30.97	94.96 ± 8.68 *	8.59 ± 1.56
SCBPE (1200 mg/kg) group	221.82 ± 13.92 **	98.02 ± 3.87 **	10.83 ± 1.61 *
SCBPE (1800 mg/kg) group	226.58 ± 16.64 **	105.96 ± 4.66 **	12.22 ± 1.89 **

^a^ Values represent mean ± SD of six animals; ^##^ Model group vs. control group, *p* < 0.01; * CDDP and SCBPE groups vs. model group, *p* < 0.05; ^**^ CDDP and SCBPE groups vs. model group, *p* < 0.01.

## Supplementary Materials

The following are available online, Uridine identification.
