# Supplementary material for: Antioxidative and Cardioprotective Effects of Schisandra chinensis Bee Pollen Extract on Isoprenaline-Induced Myocardial Infarction in Rats
Source: Molecules. 2019 Mar 20;24(6):1090. doi: 10.3390/molecules24061090 (PMC6472278; doi:10.3390/molecules24061090)
Supplement: Supplementary file 1 [file molecules-24-01090-s001.pdf]

Antioxidative and Cardioprotective Effects of *Schisandra chinensis* Bee Pollen Extract on Isoprenaline-Induced Myocardial Infarction in Rats

Zhenhuang Shen [1,2,3], Qianqian Geng [1,3], Haibo Huang [1,2,3], Hong Yao [4], Tianyu Du [1,2,3], Lifu Chen [1,3], Zhenhong Wu [1,3], Xiaoqing Miao [1,3,]* and Peiying Shi [1,3,]*

1 Bee Science College, Fujian Agriculture and Forestry University, Fuzhou 350002, China; 18065021034@163.com (Z.S.); qqgengfafu@163.com (Q.G.); haibohuang329@163.com (H.H.); tianyu1988339@sina.com (T.D.); clfbee@126.com (L.C.); wzh516@126.com (Z.W.)
2 College of Food Science, Fujian Agriculture and Forestry University, Fuzhou 350002, China
3 State and Local Joint Engineering Laboratory of Natural Biotoxins, Fujian Agriculture and Forestry University, Fuzhou 350002, China
4 Department of Pharmaceutical Analysis, School of Pharmacy, Fujian Medical University, Fuzhou 350122, China; yauhung@126.com
* Correspondence: mxqsf88@126.com (X.M.); peiyshi@fafu.edu.cn (P.S.); Tel.: +86-591-8378-9482 (P.S.)

Uridine identification

UV λ_{max}: 205, 262 nm. (–)ESI/TOF-MS *m/z* 243.06245 $[M–H]^-$. ^{1}H-NMR (CD_3OD, 400 MHz) δ ppm: 8.080 (1H, d, *J*=6.4 Hz, 6-H), 5.699 (1H, d, *J*=6.4 Hz, 5-H), 5.898 (1H, d, *J*=4 Hz, 1'-H), 4.146 (1H, dd, *J* =4, 4 Hz, 2'-H), 4.179 (1H, dd, *J* =4, 4 Hz, 3'-H), 4.099 (1H, m, 4'-H), 3.624 (2H, m, 5'-H). ^{13}C-NMR (CD_3OD) δ ppm: 164.8 (4-C), 151.1 (2-C), 141.3 (6-C), 101.3 (5-C), 89.3 (1'-C), 85.0 (4'-C), 73.5 (2'-C), 70.4 (3'-C), 61.5 (5'-C). The ^{1}H-NMR and ^{13}C-NMR profiles matched the reported NMR data for uridine [1-3].

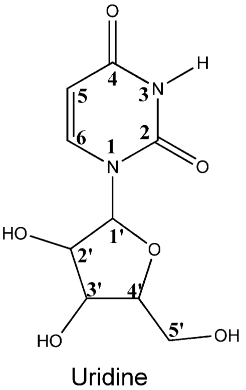

Uridine

1. Ma, L.; Zhang, Y.; Dang, J. Chemical constituents from water extract of *Armillaria luteo-virens*. *J. China Pharm. Univ.* **2016**, *47*, 291-293. Doi: 10.11665/j.issn.1000-5048.20160307.
2. Lu, H.; Xie, H.; Yang Y.; Wei, X. Chemical constituents from the macroalga *Gracilaria lemaneiformi*. *J. Trop. Subtrop. Bot.* **2011**, *19*, 166-170. Doi: 10.3969/j.issn.1005-3395.2011.02.011.
3. Mantsch, H.H.; Smith, I.C. Fourier-transformed ^{13}C NMR spectra of polyuridylic acid, uridine, and related nucleotides-the use of ^{31}POC^{13}C couplings for conformational analysis. *Biochem. Biophys. Res. Commun.* **1972**, *46*, 808-815.
